# An exploration of the views of paramedics regarding airway and resuscitation research

**DOI:** 10.1186/1757-7241-23-S2-O6

**Published:** 2015-09-11

**Authors:** Matt Thomas

**Affiliations:** 1 Great Western Air Ambulance, Bristol, UK

## Introduction and aims

Paramedics are a skilled group of clinicians with expertise in cardiac arrest. Our research group has complete a trial to comparing two supraglottic airway devices with current practice during cardiac arrest (REVIVE-Airways). This is a highly contentious topic amongst UK paramedics, The study aimed to explore the existing customs and beliefs surrounding intubation and resuscitation by UK paramedics.

## Method

We used a two level qualitative approach, conducting interviews and focus groups with paramedics. Focus groups discussed the themes arising from the interview data, developing a deeper understanding and providing insight and recommendations for future research and policy development.

### Setting

The study took place within Great Western Ambulance Service NHS Trust (GWAS). The University of the West of England, Bristol, provided sponsorship and governance. As the trial was on NHS staff ethical committee approval was not required.

### Selection & data collection

Paramedics were sampled purposefully to account for differing training and subsequently customs and beliefs and participation or not in the large trial. Supplementary snowballing was used to further identify interested/eligible paramedics. There were 34 study participants, with 17 paramedic interviews; followed by 5 focus groups with a further 17 participants. Data saturation was reached.

## Results

**Figure 1:**
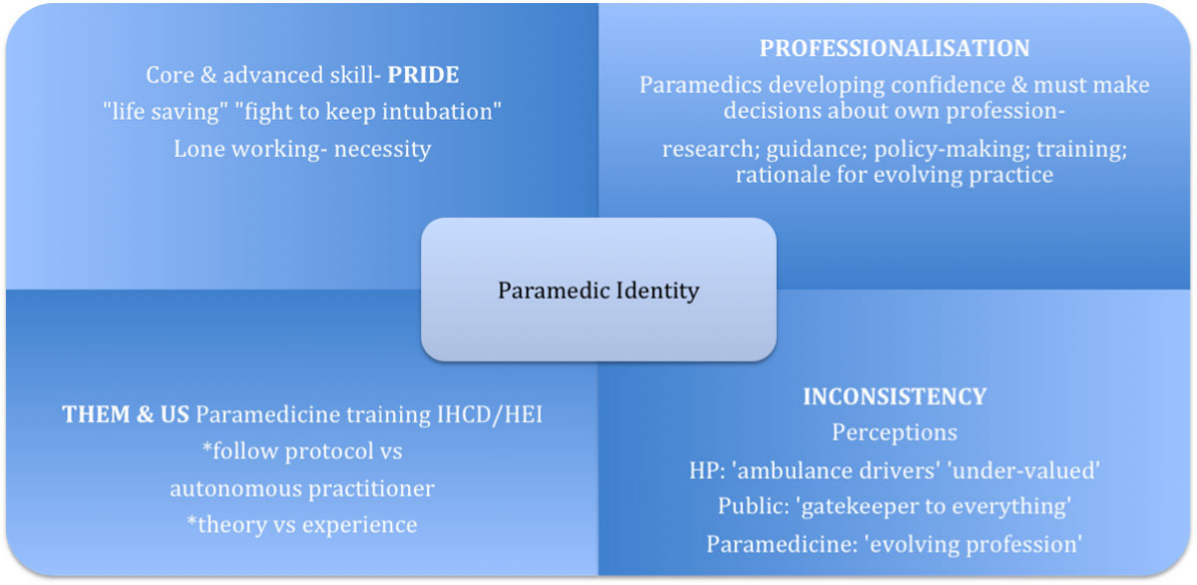
Paramedic identity

Thematic analysis was conducted. This was done in 2 stages: after interviews as a guide for focus groups, and after the focus groups. Early analysis suggests that this group of paramedics were pro-research even though some had not taken part in the earlier trial. They described four aspects of paramedic identity (figure 1.).

Specific discussion regarding intubation was focussed upon patient safety, with debate regarding the necessity of intubation in comparison to the use and success of other techniques. This stimulated concern regarding lack of training and subsequent skill fade through lack of rehearsal and competency testing. This invigorated debate about whether all paramedics should perform this task. Views differed with some vehemently protective of this skill, while others were more sanguine about this in relation to other recent skills. Frequent reference was made to the difficult situations paramedics find them selves in, specific injuries or illnesses and co-morbidities and the difficulty in retrieval of patients as a rationale for retaining the skill.

## Conclusion

Future trials in prehospital care must involve paramedics and ensure their professionalism is understood and respected.

